# Prevalence of elder abuse in elderly care centers: a review study

**Published:** 2019-08

**Authors:** Ali Nory Ghere Aghag, Parand Pourghane

**Affiliations:** ^*a*^Guilan University of Medical Sciences, Rasht, Iran.; ^*b*^Nursing, School of Nursing, Midwifery and Para-medicine, Guilan University of Medical Sciences, Rasht, Iran.

## Abstract

**Background:**

Elderly is an important public health challenge that has social, economic and health consequences. In 2016, the prevalence of anxiety was reported in 16 percent of the world’s societies, including 1 in the elderly of every 6 elder (1) According to the definition of the World Health Organization, Elder abuse is defined as any action or failure to act that results in harm to an elderly person. Elder abuse types are classified into four categories: mental, physical, sexual, and financial, and include all types of harassing Family members are official and informal carers. Environment includes communities and nursing care centers (2) in these centers, elderly patients are treated by other elderly people or carers (3). The aim of this study was to determine the prevalence of elderly people in elderly care centers.

**Methods:**

This research is a survey of the results of articles (2016-2004) that includes the evaluation of the prevalence of Elder abuse in societies and elderly caree. In order to extract information from the PubMed, PsycINFO, CINAHL, EMBASE, MEDLINE, ProQuest Criminal Justice, ASSIA databases.

**Results:**

Out of 38544 articles that have been surveyed, they determine the prevalence of Elder abuse in societies and centers of aging. Studies have shown that 64.2% of Elder abuse are executed by caretakers, which includes: 33.4% mental illness, 1 / 14% physical abuse, 13.8% financial harassment and 1.9% sexual harassment.

**Conclusions:**

According to the results, the rate of Elder abuse is high in elderly centers. In order to improve the conditions of elderly care in these centers and control of the elderly, appropriate measures and supportive policies for the elderly are needed.

**Keywords:**

Elderly, Elder abuse, Centers of Aging, Review study

